# Psychotropic medicines’ prevalence, patterns and effects on cognitive and physical function in older adults with intellectual disability in Ireland: longitudinal cohort study, 2009–2020

**DOI:** 10.1192/bjo.2023.607

**Published:** 2024-02-01

**Authors:** Marina Odalović, Ashleigh Gorman, Aviejay Paul, Philip McCallion, Éilish Burke, Malcolm MacLachlan, Mary McCarron, Martin C. Henman, Maeve Moran, Juliette O'Connell, Michael Walsh, Rohit Shankar, Caitriona Ryan, Máire O'Dwyer

**Affiliations:** School of Pharmacy and Pharmaceutical Sciences, Trinity College Dublin, Ireland; and Trinity Centre for Ageing and Intellectual Disability, Trinity College Dublin, Ireland; School of Nursing and Midwifery, Trinity College Dublin, Ireland; and Trinity Centre for Ageing and Intellectual Disability, Trinity College Dublin, Ireland; School of Social Work, College of Public Health, Temple University, Philadelphia, USA; National Clinical Programme for People with Disabilities, Health Service Executive, Dublin, Ireland; Assisting Living & Learning Institute, Maynooth University, Ireland; and Psychology Department, Maynooth University, Ireland; Faculty of Learning Disability Psychiatry, College of Psychiatrists of Ireland, Dublin, Ireland; National Clinical Programme for People with Disabilities, Health Service Executive, Dublin, Ireland; Peninsula Medical School, University of Plymouth, UK

**Keywords:** Intellectual disability, polypharmacy, antipsychotics, antidepressants, psychotropic medicines

## Abstract

**Background:**

The frequent prescribing of psychotropics and high prevalence of polypharmacy among older adults with intellectual disabilities require close monitoring.

**Aims:**

To describe change in prevalence, predictors and health outcomes of psychotropic use during the four waves (2009/2010, 2013/2014, 2016/2017, 2019/2020) of the Intellectual Disability Supplement to the Irish Longitudinal Study on Ageing (IDS-TILDA).

**Method:**

Eligible participants were adults (≥40 years) with intellectual disabilities who participated in all four waves of IDS-TILDA and who reported medication use for the entire period. Differences between groups were tested using Cochran's *Q* test for binary variables and the McNemar–Bowker test for variables with more than two categories. Generalised estimating equation models were used to assess associations between psychotropic use, participants’ characteristics and health outcomes.

**Results:**

Across waves (433 participants) there were no significant differences in prevalence of psychotropic use (61.2–64.2%) and psychotropic polypharmacy (42.7–38.3%). Antipsychotics were the most used subgroup, without significant change in prevalence between waves (47.6–44.6%). A significant decrease was observed for anxiolytics (26.8–17.6%; *P* < 0.001) and hypnotics/sedatives (14.1–9.0%; *P* < 0.05). A significant increase was recorded for antidepressants (28.6–35.8%; *P* < 0.001) and mood-stabilising agents (11.5–14.6%; *P* < 0.05). Psychotropic polypharmacy (≥2 psychotropics) was significantly associated with moderate to total dependence in performing activities of daily living over the 10-year period (OR = 1.80, 95% CI 1.21–2.69; *P* < 0.05).

**Conclusions:**

The study indicates an increase in usage of some classes of psychotropic, a reduction in others and no change in the relatively high rate of antipsychotic use over 10 years in a cohort of older adults with intellectual disabilities and consequent risk of psychotropic polypharmacy and medication-related harm.

Adults with intellectual disability are frequently prescribed psychotropic medicines.^1–4^ This may be beneficial for the treatment of mental health conditions, but inappropriate prescribing and increased age-related sensitivity to these medicines increase the risk of medication-related harm such as falls, sedation and constipation.^5,6^ The practice of ‘off-label’ prescribing in this cohort is widely debated and is of international concern.^1,7^ In addition, the increased medical needs of adults with intellectual disability compared with the general population, and the lack of supports for this population, result in a poorer health status.^8,9^ Pooled prevalence reported in a recent systematic review suggested the following extent of psychotropic use in adults with intellectual disability: any psychotropic: 41% (95% CI 35–46%); antipsychotics: 31% (95% CI 27–35%); antidepressants: 14% (95% CI 9–19%); anxiolytics: 9% (95% CI 4–15%); hypnotics/sedatives: 5% (95% CI 2–8%); and psychostimulants: 1% (95% CI 1–2%).^4^ Additionally, psychotropic polypharmacy (the concurrent use of two or more psychotropics^2^) is common in adults with intellectual disability, with pooled prevalence of 17–40%.^4^ Given the lack of prescribing guidance in older adults with intellectual disabilities, the high number of older adults prescribed psychotropics (including antipsychotics) without a doctor's diagnosis of psychosis and the high level of psychotropic polypharmacy, it is important to examine the extent and trends of psychotropic use over time in a cohort of older adults with intellectual disability and the impact of these medications.

## Method

### Aim

The aim of this study is to examine the change in prevalence and predictors of psychotropic drug prescription among adults with intellectual disability who participated in all four waves of the Intellectual Disability Supplement to the Irish Longitudinal Study on Ageing (IDS-TILDA) between 2009 and 2020. Secondarily, the study will investigate the effects of psychotropic drugs on cognitive and physical function over time. This work is part of a larger study, Examining Quality, Use and Impact of Psychotropic (use) in older adults with intellectual disabilities (EQUIP), whose study protocol has been published elsewhere.^10^

### Study design

This a cohort study using the data on participant characteristics, psychotropic use, and physical and cognitive health outcomes of participants in all four waves of IDS-TILDA with valid medicines data: Wave 1 (2009/2010), Wave 2 (2013/2014), Wave 3 (2016/2017) and Wave 4 (2019/2020). IDS-TILDA is a longitudinal study of adults with intellectual disability aged ≥40 years and has been described in detail elsewhere.^11–13^ Data were collected by the pre-interview questionnaire (PIQ) and during the face-to-face computer-assisted personal interview (CAPI) at all four waves. The CAPI was completed by a trained interviewer using a combination of self-reported, supported and proxy interviews. The majority were completed by the participants themselves or they were supported in doing so by a person of their choosing. In some cases, a proxy who had known and worked with the participant for at least 6 months completed on the participant's behalf.^11^ At all four waves, IDS-TILDA had ethical approval from the Faculty of Health Sciences Research Ethics Committee at Trinity College Dublin and 138 intellectual disability service providers. If participants were able to understand the accessible easy-read information booklet, consent was obtained directly from them. In Wave 4, consent declaration was in place from the Health Research Consent Declaration Committee for participants who lacked capacity to consent. For participants who lacked capacity to provide consent themselves, assent was obtained from a proxy (e.g. family member/guardian). Consent was reaffirmed at data collection and throughout the participants’ interviews or objective health measures appointments at all four waves. Participants’ rights to withdraw at any time were upheld. The Strengthening the Reporting of Observational Studies in Epidemiology (STROBE) reporting guidelines for cohort studies was utilised.

### Ethics statement

The authors assert that all procedures contributing to this work comply with the ethical standards of the relevant national and institutional committees on human experimentation and with the Helsinki Declaration of 1975, as revised in 2008. All procedures involving human subjects/patients were approved by the Faculty of Health Sciences Research Ethics Committee at Trinity College Dublin and 138 intellectual disability service providers.

### Study cohort

The study sample was generated from the IDS-TILDA population. Inclusion criteria were participation in all four waves and medication data available for the entire study period.

IDS-TILDA is a large-scale, nationally representative study of people aged 40 years and over with an intellectual disability in Ireland. A representative sample of 1800 people with intellectual disability was drawn from the National Intellectual Disability Database. Of these, a total of 753 individuals, representing 8.9% of the intellectual disability population ≥40 years of age, consented to take part in the study in Wave 1.^11^ The geographical representativeness of the sample was examined by plotting the locations of everyone interviewed.^11^ The 753 people successfully recruited were also examined in terms of representativeness of geographical subgroups, as follows: (a) ‘living in Dublin city or county’, 28.1%; (b) ‘living in a town or city in the Republic of Ireland’, 55.8%; and (c) ‘living in a rural area in the Republic of Ireland’, 16.1%.^11^ All ten Health Service Executive (HSE) areas were found to be represented.^11^ After exclusion of those who withdrew (*n* = 75) or who died (*n* = 172), the sample involved 506 participants active in all four waves.^14^ Participants who did not provide medication data in at least one wave (*n* = 73) were excluded. The final study sample included 433 participants. Supplementary Appendix 1, available at https://doi.org/10.1192/bjo.2023.607, presents a flowchart for the study.

### Medication data

Information on the medication data were obtained in the PIQ and the answers were confirmed in the CAPI at all four waves. Details on the gathering, recording and ensuring high quality and accuracy of the medication data are published elsewhere.^2^

The following Anatomical Therapeutic Chemical (ATC) drug classes were observed as psychotropic medicines:

- N05A: antipsychotics
- N05B: anxiolytics
- N05C: sedatives/hypnotics
- N06A: antidepressants
- anti-seizure medications (N03A) reported by people without a diagnosis of epilepsy and lithium (N05AN01): mood stabilising agents.

Several drug reclassifications were undertaken on reviewing the main clinical use of the drug, in line with previous studies:^2,3^ lithium as a mood stabiliser; prochlorperazine as an anti-emetic/anti-nauseant; clonazepam as an anxiolytic in participants who had no diagnosis of epilepsy and reported diagnosis of a mental health condition; rectal diazepam and clobazam were removed from anxiolytics; midazolam was removed from the hypnotic/sedative subclass.^2^

The following variables were created after reclassifications to maintain comparability with other studies:^2,3^

- six binary variables reporting use of (i) any psychotropic, (ii) antipsychotic, (iii) anxiolytic, (iv) sedative/hypnotic, (v) mood-stabilising agent and (vi) antidepressant;
- six categorical variables reporting 0, 1 or 2+ psychotropics (psychotropic polypharmacy) for each drug subcategory:^2^ (i) any psychotropic, (ii) antipsychotics, (iii) anxiolytics, (iv) sedatives/hypnotics, (v) mood-stabilising agents and (vi) antidepressants;
- three binary variables reporting interclass psychotropic polypharmacy (i.e. taking at least one medication from each of observed psychotropic subclasses): (i) antipsychotics, anxiolytics, sedatives/hypnotics, (ii) antipsychotics, anxiolytics, sedatives/hypnotics, antidepressants, (iii) antipsychotics, antidepressants;^2^
- six count variables reporting total number of medicines per person for each drug subcategory: (i) any psychotropic, (ii) antipsychotic, (iii) anxiolytic, (iv) sedative/hypnotic, (v) mood-stabilising agent and (vi) antidepressant.

### Physical and cognitive health outcomes

Several health outcomes were identified in the literature and baseline cross-sectional IDS-TILDA studies as potential adverse outcomes associated with psychotropic drug use.^2,13^ The following outcomes were analysed to assess association between the observed outcomes and psychotropic use across the waves (further information, such as questioning in the original scale and the modifications undertaken, is provided in Supplementary Appendices 2–4):

- functional status, assessed using the Barthel Index (Supplementary Appendix 3) scores: total dependence (score of 0–4), severe dependence (5–12), moderate dependence (13–18), mild dependence (19) and total independence (20);^15^ a binary variable was created for statistical purposes, with the following categories: ‘mild dependence/total independence’ and ‘moderate/severe/total dependence’;
- chronic constipation (yes/no): reported doctor's diagnosis of chronic constipation;
- falls in the past year (yes/no): any fall, including a slip or trip, in the previous year;
- dementia (yes/no)**:** reported doctor's diagnosis of Alzheimer's disease, dementia, organic brain syndrome or senility and serious memory impairment, as well as reporting of any anti-dementia drug (identified using the ATC code N06D).

### Covariates

#### Demographics

The following characteristics were considered as demographic covariates: age (40–49; 50–64; 65+ years), gender (male; female), type of residence (independent; community group home; residential care), reported level of intellectual disability (mild; moderate; severe/profound). Independent living is considered as living either independently or semi-independently, and living in the family home; community group home is defined as a community setting with staff support for small groups of people with intellectual disability; residential care represents living arrangements where 10+ people share a single living unit or where the living arrangements are campus based.^2,16^

#### Health

Several health-related covariates were observed (more details in Supplementary Appendix 2):

- ability to walk 100 yards: level of difficulty with walking 100 yards; a binary variable was created with the following categories: ‘no/some difficulty’ and ‘a lot of difficulty/cannot do at all’;
- epilepsy (yes/no): reported doctor's diagnosis of epilepsy as a historical diagnosis;
- mental health condition (yes/no): reported doctor's diagnosis of an emotional, nervous or psychiatric condition (hallucinations, anxiety, depression, emotional problems, schizophrenia, psychosis, mood swings, manic depression, post-traumatic stress disorder, other);
- usage of opioids (yes/no): medicines belonging to N02A class were considered as opioids; in waves 1–3 paracetamol combinations with codeine were coded as N02BE51 and so this was included in the opioid count in waves 1–3; opioids were analysed in line with constipation as it is a common side-effect.

### Statistical analysis

Descriptive statistics of population characteristics were calculated as proportions of the total study sample (*n* = 433). For discrete variables, medians and interquartile ranges (IQRs) were reported as the data were not normally distributed.

Differences in proportions between the four waves were tested using Cochran's *Q* test for paired samples of dichotomous variables (e.g. psychotropic drug use: yes/no) with *post hoc* analysis for pairwise comparisons to reveal which specific waves differed from each other. The McNemar–Bowker test was used to test differences in proportions between waves in the case of categorical variables with three categories (e.g. psychotropic polypharmacy: 0/1/2+ medicines). The results of the McNemar–Bowker test indicated which categories significantly differed from each other and which time points were significantly different. The Holm–Bonferroni correction was applied to control for type I errors (false-positive results) and type II errors (false-negative results).

The generalised estimating equations (GEE) procedure extends the generalised linear model to analyse repeated measures.^17^ GEE models were used to estimate the associations between psychotropic drug use and participants’ demographic and health-related characteristics across the four time points over the 10 years. The GEE models included the main effects of independent variables and handled missing data. Based on the type of dependent variable, binary or count, models were specified to use a binomial or Poisson distribution respectively. Odds ratios (OR) and the corresponding 95% confidence intervals (95% CI) were reported. A correlation matrix representing the within-participant dependencies is estimated as part of the model. Since the same cohort of participants was observed over time, with data collected in equal time periods over 10 years, the following correlation matrices were considered as the most appropriate, and accordingly used within GEE models: autoregressive (assumes that observations that are closer together in time have a higher correlation than those further apart, i.e. the correlation between two observations decreases over time) and exchangeable (assumes that the correlation between any two observations within the same cluster is the same).^18^ Decision between autoregressive and exchangeable correlation matrix was defined based on quasi-likelihood information criterion (QIC) since the more commonly used Akaike information criterion (AIC) cannot be used here as GEE is not a likelihood-based method. A lower QIC value indicates a better model fit. Data analyses were performed using R for Windows, version 4.2.2^19,20^ and SPSS for Windows, version 27.^21^ Statistical significance was set at *P* < 0.05.

## Results

**Fig. 1:**
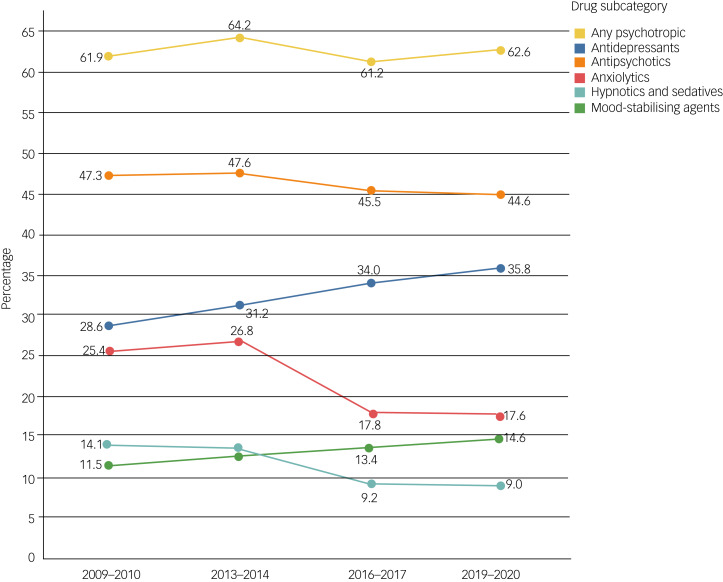
The change in prevalence of psychotropic prescribing across the 10-year period 2009/2010–2019/2020 (*n* = 433 participants).

Participants’ characteristics and their changes over the study period are presented in Table 1. Since the same cohort was observed over 10 years, participant age was significantly higher in later waves in comparison with earlier (*P* < 0.001). Additionally, a significant change was observed in residence, where an increase in those living in community group homes and decrease in those living in a residential care setting was recorded between Wave 1 and Wave 2, and Wave 1 and Wave 4 (*P* < 0.05). Over the waves the study cohort also reported an increasing prevalence of severe difficulty in walking 100 yards, moderate/severe/total dependence in performing activities of daily living and chronic constipation (*P* < 0.001) and having epilepsy (*P* < 0.05) (Table 1). Although overall there were no significant differences in trends and prevalence of reported mental health conditions between waves (Table 1), there was a notable decrease in the prevalence of depression between Wave 1 (21.0%) and Wave 4 (12.2%) (*P* < 0.05), as well as an increase in prevalence of emotional problems between Wave 2 (11.1%) and Wave 4 (18.1%) (Supplementary Appendix 5). However, the use of antidepressants increased between Wave 1 (28.6%) and Wave 4 (35.8%) (Fig. 1). Data from IDS-TILDA do not allow us to make a definitive link between diagnoses and medicines, as reason for prescribing is not collected. No significant change in other participant characteristics was observed (*P* > 0.05).

**Table 1:** Participants’ characteristics over the 10-year period 2009/10–2019/20 (*n* = 433)

Characteristic	Wave 1, *n*; %	Wave 2, *n*; %	Wave 3, *n*; %	Wave 4, *n*; %	Difference in the proportions between waves, *P*^a^
Age, years					<0.001^b^
40–49	185; 42.7	127; 29.3	47; 10.9	–	
50–64	200; 46.2	239; 55.2	286; 66.1	276; 63.7	
65+	48; 11.1	67; 15.5	100; 23.1	157; 36.3	
Gender					>0.05
Male	187; 43.2	187; 43.2	187; 43.2	187; 43.2	
Female	246; 56.8	246; 56.8	246; 56.8	246; 56.8	
Residence					**<0.05**^c^
Independent/Family	61; 14.1	64; 14.8	58; 13.4	49; 11.4	
Community group home	168; 38.8	194; 44.8	182; 42.0	202; 47.1	
Residential care	204; 47.1	175; 40.4	193; 44.6	178; 41.5	
Missing	*n* = 0	*n* = 0	*n* = 0	*n* = 4	
Level of intellectual disability					>0.05
Mild	94; 23.6	94; 23.6	94; 23.6	102; 24.6	
Moderate	182; 45.6	182; 45.6	182; 45.6	189; 45.5	
Severe/profound	123; 30.8	123; 30.8	123; 30.8	124; 29.9	
Missing	*n* = 34	*n* = 34	*n* = 34	*n* = 18	
Walking 100 yards					**<0.001** Wave 1 *v.* Wave 4 Wave 2 *v.* Wave 4 Wave 2 *v.* Wave 3
No/some difficulty	373; 86.3	351; 81.4	339; 78.3	299; 72.9	
A lot of difficulty/ cannot do at all	59; 13.7	80; 18.6	94; 21.7	111; 27.1	
Missing	*n* = 1	*n* = 2	*n* = 0	*n* = 23	
Epilepsy					***P* < 0.05** Wave 1 *v.* Wave 3 Wave 2 *v.* Wave 3
No	290; 67.4	290; 67.6	276; 63.9	282; 65.1	
Yes	140; 32.6	139; 32.4	156; 36.1	151; 34.9	
Missing	*n* = 3	*n* = 4	*n* = 1	*n* = 0	
Chronic constipation					**<0.001** Wave 1 *v.* Wave 2 Wave 1 *v.* Wave 3 Wave 1 *v.* Wave 4 Wave 2 *v.* Wave 4
No	356; 82.2	270; 62.9	241; 55.8	215; 49.7	
Yes	77; 17.8	159; 37.1	191; 44.2	218; 50.3	
Missing	*n* = 0	*n* = 0	*n* = 1	*n* = 0	
Falls in previous year					>0.05
No	308; 71.5	308; 72.3	306; 71.2	292; 68.2	
Yes	123; 28.5	118; 27.7	124; 28.8	136; 31.8	
Missing	*n* = 2	*n* = 7	*n* = 3	*n* = 5	
Mental health condition					>0.05
No	196; 47.0	196; 47.0	195; 45.1	199; 46.0	
Yes	221; 53.0	221; 53.0	237; 54.9	234; 54.0	
Missing	*n* = 16	*n* = 16	*n* = 1	*n* = 0	
Dementia & Alzheimer's disease					>0.05
No	417; 96.3	418; 96.5	409; 94.5	406; 93.8	
Yes	16; 3.7	15; 3.5	24; 5.5	27; 6.2	
Barthel Index					***P* < 0.001** Wave 1 *v.* Wave 4 Wave 2 *v.* Wave 4
Mild dependence/total independence	133; 31.5	111; 27.1	109; 25.4	80; 19.3	
Moderate/severe/total dependence	289; 68.5	298; 72.9	320; 74.6	335; 80.7	
Missing	*n* = 11	*n* = 24	*n* = 4	*n* = 18	
Opioid drug use					*P* > 0.05
No	420; 97.0	418; 96.5	422; 97.5	412; 95.2	
Yes	13; 3.0	15; 3.5	11; 2.5	21; 4.8	

a. Specific waves that significantly differed from each other in pairwise comparisons are presented in the table; *post hoc* analysis on differences between each two categories across waves.

b. Age (years): 40–49 *v.* 50–64, across each pair of waves, *P* < 0.001; 50–64 *v.* 65+ across each pair of waves, *P* < 0.001.

c. Residence: Community group home *v.* Residential care, Wave 1 *v.* Wave 2, Wave 1 *v.* Wave 4, *P* < 0.05.

### Change in psychotropic prevalence over time

There was no significant difference in use of any psychotropic over the observed 10-year period (Wave 1: 61.9%; Wave 4: 62.6%) (Supplementary Appendix 6). However, a significant decrease over the waves was observed for anxiolytic (Wave 1: 25.4%; Wave 4: 17.6%; *P* < 0.001) and hypnotic/sedative drugs (Wave 1: 14.1%; Wave 4: 9.0%; *P* < 0.001). An increase was observed for antidepressants (Wave 1: 28.6%; Wave 4: 35.8%; *P* < 0.001) and mood-stabilising agents (Wave 1: 11.5%; Wave 4: 14.6%; *P* < 0.05) (Supplementary Appendix 6).

Use of some individual psychotropics decreased: diazepam (Wave 1: 15.7%; Wave 4: 6.5%; *P* < 0.001), chlorpromazine (Wave 1: 10.6%; Wave 4: 6.2%; *P* < 0.001) and lorazepam (Wave 1: 9.5%; Wave 4: 3.7%; *P* < 0.001) (Supplementary Appendix 7). However, the use of the following drugs increased over the time: sertraline (Wave 1: 3.0%; Wave 4: 9.9%; *P* < 0.001) and quetiapine (Wave 1: 2.3%; Wave 4: 5.8%; *P* < 0.001) (Supplementary Appendix 7).

### Psychotropic intraclass and interclass polypharmacy

Examination of psychotropic intraclass polypharmacy (use of two or more agents from the same therapeutic class) shows no significant change in prevalence of any psychotropic intraclass polypharmacy over the observed period (Supplementary Appendix 8).

Psychotropic interclass polypharmacy decreased for the following combinations: (a) antipsychotic, anxiolytic and sedative/hypnotic (Wave 1: 6.2%; Wave 4: 0.9%; *P* < 0.05), (b) antipsychotic, anxiolytic, sedative/hypnotic and antidepressant (Wave 1: 4.2%; Wave 4: 0.5%; *P* < 0.05) (Supplementary Appendix 9). There was no significant change in prevalence of antipsychotic and antidepressant combination over time (Wave 1: 20.8%; Wave 2: 21.7%; Wave 3: 22.9%; Wave 4: 22.6%; *P* > 0.05) (Supplementary Appendix 9).

### Factors and outcomes associated with psychotropic use over time

GEE models using binomial distribution (dependent variable: psychotropic use ‘yes/no’ (binary variable)) revealed factors significantly associated with psychotropic use over the 10-year period: age (associated with anxiolytic use), gender (associated with antidepressant use), residential setting (associated with any psychotropic, antipsychotic, anxiolytic and antidepressant use), more severe level of intellectual disability (associated with hypnotic/sedative and antidepressant use), epilepsy (associated with anxiolytic, mood-stabilising agent and antidepressant use), mental health condition (associated with any psychotropic use and all psychotropic subclasses), time (associated with anxiolytic, hypnotic/sedative, mood-stabilising agent and antidepressant use) (Table 2). Additionally, the GEE model using Poisson distribution (dependent variable: number of psychotropics (count variable)) demonstrated significantly higher expected number of psychotropics for those who lived in residential care (OR = 1.46, 95% CI 1.09–1.96), reported severe/profound level of intellectual disability (OR = 1.29, 95% CI 1.02–1.65) and mental health condition (OR = 1.65, 95% CI 1.44–1.89) (Supplementary Appendix 10).

**Table 2:** Factors associated with psychotropic use by drug class for the study cohort (*n* = 433)

Characteristic	Any psychotropic, OR (95% CI)	Antipsychotics, OR 95% CI)	Anxiolytics, OR (95% CI)	Hypnotics/sedatives, OR (95% CI)	Mood-stabilising agents, OR (95% CI)	Antidepressants, OR (95% CI)
Age, years						
40–49	1	1	1	1	1	1
50–64	0.82 (0.64–1.07)	1.00 (0.81–1.23)	0.77 (0.59–1.07)	0.89 (0.66–1.20)	0.89 (0.53–1.50)	0.96 (0.75–1.23)
65+	1.13 (0.76–1.67)	1.11 (0.80–1.54)	0.61 (0.37–1.01)**	1.02 (0.61–1.70)	1.16 (0.58–2.31)	0.81 (0.56–1.18)
Gender						
Male	1	1	1	1	1	1
Female	1.19 (0.85–1.67)	0.81 (0.58–1.13)	0.76 (0.53–1.09)	1.27 (0.76–2.11)	0.69 (0.30–1.62)	1.81 (1.24–2.64)*
Residence						
Independent/family	1	1	1	1	1	1
Community group home	2.55 (1.62–4.03)*	1.61 (1.07–2.43)*	2.93 (1.37–6.29)*	1.18 (0.66–2.10)	1.20 (0.45–3.25)	1.52 (0.96–2.42)
Residential care	2.74 (1.78–4.23)*	1.90 (1.29–2.81)*	3.70 (1.74–7.87)*	1.25 (0.69–2.26)	1.75 (0.64–4.75)	1.66 (1.02–2.71)*
Level of intellectual disability						
Mild	1	1	1	1	1	1
Moderate	0.76 (0.49–1.19)	1.03 (0.67–1.58)	1.63 (0.93–2.83)	1.94 (0.88–2.43)	3.09 (0.55–17.53)	0.75 (0.48–1.17)
Severe/profound	0.86 (0.53–1.41)	1.46 (0.92–2.34)	1.20 (0.68–2.14)	2.18 (1.01–4.73)*	2.75 (0.39–19.32)	0.53 (0.32–0.90)*
Epilepsy						
No	1	1	1	1	1	1
Yes	0.83 (0.60–1.13)	0.74 (0.54–1.02)	1.64 (1.18–2.30)*	1.54 (0.98–2.42)	0.03 (0.00–0.74)*	0.63 (0.46–0.86)*
Mental health condition						
No	1	1	1	1	1	1
Yes	5.08 (3.80–6.78)*	2.51 (1.93–3.27)*	2.53 (1.83–3.51)*	1.75 (1.21–2.53)*	2.55 (1.28–5.11)*	2.09 (1.59–2.75)*
Time						
Wave 1 (2009/2010)	1	1	1	1	1	1
Wave 2	1.14 (0.95–1.37)	1.01 (0.89–1.15)	1.16 (0.88–1.54)	1.01 (0.77–1.33)	1.29 (0.89–1.86)	1.10 (0.94–1.31)
Wave 3	0.93 (0.76–1.14)	0.91 (0.77–1.09)	0.62 (0.45–0.86)*	0.63 (0.44–0.91)*	1.61 (1.09–2.39)*	1.32 (1.10–1.60)*
Wave 4 (2019/2020)	1.00 (0.77–1.29)	0.86 (0.69–1.06)	0.68 (0.47–0.99)*	0.64 (0.42–0.98)*	1.60 (0.99–2.58)	1.49 (1.18–1.87)*

* *P* < 0.05, **P = 0.05; binary dependent variable: Not taking drug (reference category)/Taking drug.

As shown in Table 3, psychotropic polypharmacy was significantly associated with moderate/severe/total dependence in performing activities of daily living over the 10-year period, after adjusting for demographic and health-related confounders (OR = 1.80, 95% CI 1.21–2.69; *P* < 0.05). Psychotropic polypharmacy was not associated with falls, chronic constipation or dementia.

**Table 3:** The association of health-related outcomes and psychotropic drug use, controlled for health and participants’ (*n* = 433) demographic characteristics

Characteristic	Falls: not reported (reference)/reported, OR (95% CI)	Chronic constipation: not reported/(reference), reported, OR (95% CI)	Barthel index: mild dependence/total independence (reference)/moderate/severe/total dependence, OR (95% CI)	Dementia: not reported (reference)/ reported, OR (95% CI)
Any psychotropic use				
None	1	1	1	1
1	0.96 (0.67–1.37)	1.19 (0.84–1.68)	1.41 (0.93–2.14)	0.84 (0.41–1.73)
2+	0.97 (0.69–1.36)	1.26 (0.91–1.75)	1.80 (1.21–2.69)*	1.54 (0.70–3.40)
Age (years)				
40–49	1	1	1	1
50–64	1.48 (1.07–2.04)*	0.86 (0.62–1.19)	1.27 (0.88–1.83)	1.12 (0.58–2.17)
65+	1.82 (1.18–2.83)*	1.14 (0.72–1.79)	2.68 (1.57–4.58)*	1.31 (0.55–3.12)
Gender				
Male	1	1	1	1
Female	1.08 (0.80–1.45)	1.33 (0.96–1.83)	1.50 (1.04–2.15)*	0.82 (0.42–1.63)
Residence				
Independent/family	1	1	1	1
Community group home	1.24 (0.79–1.94)	2.15 (1.15–4.03)*	1.50 (0.94–2.38)	–
Residential care	1.55 (0.97–2.46)	2.49 (1.28–4.84)*	3.14 (1.87–5.26)*	–
Level of intellectual disability				
Mild	1	1	1	1
Moderate	1.08 (0.75–1.55)	1.69 (1.10–2.59)*	1.95 (1.30–2.92)*	1.08 (0.44–2.65)
Severe/profound	0.67 (0.43–1.05)	2.98 (1.83–4.87)*	15.82 (8.04–31.13)*	0.75 (0.27–2.07)
Epilepsy				
No	1	1	1	1
Yes	1.52 (1.12–2.06)*	1.64 (1.18–2.26)*	1.17 (0.78–1.76)	2.71 (1.29–5.68)*
Mental health condition				
No	1	1	1	1
Yes	1.39 (1.04–1.86)*	1.64 (1.18–2.26)*	1.39 (0.99–1.93)	1.18 (0.62–2.27)
Walking 100 yards				
No/some difficulties	1	1	–	–
A lot of difficulties, cannot do at all	1.04 (0.75–1.46)	1.37 (0.99–1.91)	–	–
Time				
Wave 1 (2009/2010)	1	1	1	1
Wave 2	0.91 (0.69–1.20)	2.87 (2.16–3.81)*	1.12 (0.82–1.51)	0.80 (0.42–1.53)
Wave 3	0.91 (0.67–1.22)	4.02 (2.85–5.66)*	1.09 (0.79–1.49)	1.32 (0.70–2.47)
Wave 4 (2019/2020)	0.93 (0.67–1.29)	5.03 (3.50–7.23)*	1.58 (1.09–2.77)*	1.59 (0.77–3.29)
Opioid drug use				
No	–	1	–	–
Yes	–	1.55 (0.91–2.64)	–	–

* *P* < 0.05; Dependent variables: Falls (Does not have fall in previous year (reference category)/Had fall in previous year), Chronic constipation (Does not have (reference category)/Has).

## Discussion

Psychotropic use remained commonplace among older adults with intellectual disability in Ireland over 10 years, with over six in ten of the cohort reporting at least one psychotropic at all four time points (between 2009/2010 and 2019/2020). TILDA reported that 15% of its general population participants aged ≥65 were taking ≥1 psychotropic medication at Wave 1 of the study (2009/2011),^22^ meaning that the finding of around 60% in IDS-TILDA highlights major differences in the extent of psychotropic use between these two populations. Psychotropic polypharmacy was also higher, with almost four in ten reporting use of two or more psychotropics at all four time points. Antipsychotics remained the most commonly used, reported by at least 44.6% of the cohort at all four waves. Some significant changes in patterns of psychotropic use were evident, with a decrease in anxiolytic and hypnotic/sedative use and a significant increase in antidepressant use. There was a significant change in use of several individual drugs: increase in newer generation antidepressants (sertraline) and antipsychotics (quetiapine) and decrease of the first-generation antipsychotics (chlorpromazine). Similar changes and an increase in sertraline and quetiapine use were also recorded in studies in the UK and Canada.^23–25^ A significant decrease in the use of the most prevalent interclass polypharmacy combination in Wave 1 – antipsychotic/anxiolytic/sedative/hypnotic – was observed. Psychotropic polypharmacy was revealed as independently associated with moderate to total dependence in performing activities of daily living over time after adjusting for confounders, but no significant association was found between psychotropic use and other healthcare outcomes over the 10 years.

People with intellectual disability may be more likely to be placed in residential settings if they have dementia and co-occurring conditions,^26^ which potentially can lead to the prescription of more medication. This may in part be due to inequity in access to adequate mental health residential places or day services. In addition, social inclusion may not always be facilitated in community group homes owing to insufficient provision of multi-disciplinarity teams or insufficient attention given to activities that promote social inclusion. Further studies are needed to reveal reasons for relatively high psychotropic use across a decade among adults with intellectual disability in Ireland.

Other studies which observed similar cohorts of people with intellectual disability over time have shown an increase in psychotropic drug use (Supplementary Appendix 11) in Australia (43.3–54.2%, 1999–2015) and Scotland (47.0–57.8%, 2002–2014).^27,28^ In comparison with Australia and Scotland, a higher and stable prevalence of psychotropic drug use over time was recorded in our cohort of adults with intellectual disability in Ireland (61.9–62.6%, 2009/2010–2019/2020). The differences in extent of psychotropic drug use may reflect different prevalence of mental health conditions (53–54% in Ireland versus 29.3% in Australia^27^ and 36.8% in Scotland^28^) and the reasons for the higher prevalence in Ireland should be identified. Psychotropic prevalence rates might also be affected by different groups of drugs classified as psychotropics. Antipsychotics, anxiolytics, hypnotics/sedatives and antidepressants were included in all the above-mentioned studies. However, the Australian study also included psychostimulants, anti-Parkinsonian medications and opioid antagonists, and the Scottish study included anti-epileptics. The narrower inclusion criteria in this Irish study, associated with a higher coverage of psychotropic use, suggests our results may be a conservative reflection of psychotropic prescription in Ireland compared with other countries.

Change of prescribing patterns reflected by the increased use of antidepressants and a decreasing or stable trend in antipsychotic use was reported in several previous studies among people with intellectual disability. An increase in antidepressant use between 1999 and 2015 in Australia (16.7 *v.* 36.1%) and a stable use of antipsychotics (26.7 *v.* 27.7%) were recorded. Accordingly, antidepressants had become the most used psychotropics at the end of the observed period^27^ (Supplementary Appendix 11). Several studies in England reported an increasing trend in antidepressant use over the past decade, with the most recent study undertaken for 13 years. That study reported a rise in antidepressant prescribing rates among people with intellectual disability, from 16.9% (2009–2012) to 24.6% (2021).^29^ A TILDA report highlights relatively stable trends of antidepressant use among the general population, from 6.97 to 10.04%, over 6 years (2010–2016),^30^ whereas our findings from IDS-TILDA show increases in antidepressant use from 28.6% (2009/2010) to 35.8% (2019/2020). The suggestion here of significantly higher antidepressant usage among adults with intellectual disability in comparison with the general older population in Ireland deserves further investigation.

Given the decrease in the prevalence of depression, an increase in antidepressant use seems contradictory, however the increase in the reporting of emotional problems must be noted. Possible explanations for the higher rate of antidepressant use among older people with intellectual disability may include that adults with a cognitive or self-care disability often interact more with healthcare professionals, who therefore recognise symptoms of depression more frequently than in the general population.^31^ Additionally, social restrictions implemented during the COVID-19 pandemic were associated with a rise in the diagnosis of depression: during the first year of the pandemic (2020) the number of prescriptions of antidepressants among the general population in England increased by 4 million.^23^ The potential contribution of the COVID-19 pandemic to the increase of antidepressant usage among adults with intellectual disability in Ireland should be studied in detail in further studies.

This study has confirmed minimal change in the relatively high prevalence of antipsychotic use (from 47.3% to 44.6%) between 2009/2010 and 2019/2020. The reported prevalence is still higher in comparison with other countries: for example England^29^ reported 17–18% during 2009/2012–2021 (Supplementary Appendix 11). Despite the trend of increasing antipsychotic use among the general population, the extent of antipsychotic use is quite low among people living in private households in England: 0.6% (95% CI 0.4–0.7%) in 2000 and 1.2% (95% CI 0.9–1.5%) in 2014.^29^ Such findings raise concerns about long-term and extensive antipsychotic prescribing in persons with intellectual disability in Ireland. Reasons for decisions not to discontinue antipsychotics and consequent long-term antipsychotic use have been reported in the literature, including concerns about feelings of restlessness, the presence of an autism spectrum disorder, previously unsuccessful attempts to discontinue and objections against discontinuation by legal representatives.^32^ However, regular review of psychotropic side-effects, particularly in people with psychotropic polypharmacy, has been recognised as essential for clinical decision-making about (dis)continuation and can be helpful in resolving clinicians’ concerns.^33^ A range of psychosocial therapeutic and supportive interventions, including social prescribing, are often necessary to facilitate psychotropic deprescribing. Without alternatives clinicians may have few options other than to continue prescribing psychotropics for people who continue to experience mental health problems.

This study revealed a significant declining trend of anxiolytic and hypnotic/sedative use among adults with intellectual disability in Ireland. This finding indicates a substantial change in prescribing patterns for these groups of drugs. Age was revealed as significant predictor of lower anxiolytic usage: over the 10-year period, participants aged 65+ years had an almost 40% lower odds of using anxiolytics compared with those aged 40–49 years. Such changes may also reflect recommendations of HSE guidance on appropriate prescribing of benzodiazepines and Z-drugs for the treatment of anxiety and insomnia issued in 2018,^34^ and may illustrate the effects that clear policy changes can have on prescribing practices.

Only a small number of studies have explored factors associated with psychotropic drug use among adults with intellectual disabilities. Significant associations between psychotropic use, living in residential settings and the presence of mental health conditions, together with an association between psychotropic polypharmacy and the absence of epilepsy, have been reported.^2,3^ The present study supports these previous results and identifies additional specific factors significantly associated with subcategories of psychotropic. Female gender was significantly associated with more antidepressant usage over time, age was significantly associated with more anxiolytic use over time and level of intellectual disability was significantly associated with more hypnotic/sedative use over time. A significant increase in constipation prevalence over time was recorded. However, although constipation is stated as a side-effect of many psychotropic drugs, the association between psychotropic drug use and constipation in this cohort has not been confirmed. Moreover, an association was found between use of two or more psychotropics and greater dependence in performing activities of daily living. These demographic and clinical variables should be taken into account in efforts to understand prescribing practices and optimise the prescribing of psychotropic drugs to people with intellectual disability. Our finding that having a fall in the previous year was not significantly associated with psychotropic drug use across the 10-year period must be interpreted conservatively, given that it is in contrast to studies in the general older population.^13,34,35^ There are higher rates of medicine use overall and the complications of existing disabilities in the study population. Perhaps the picture is too complex in this population to discern specific contributions of psychotropic polypharmacy. The issue deserves further consideration.

### Clinical implications

The decrease in the use of the anxiolytic and hypnotic and sedative classes of psychotropic seems a positive change and may be associated with system-wide measures being taken during the study period to reduce the use of these drugs. The increased prescription of antidepressant medication needs to be further investigated longitudinally, together with the association of antidepressant use and female gender and whether antidepressants are substituting for anxiolytics.^25^ Antidepressant prescribing may be appropriate in a population whose vulnerability and experience of psychological trauma is often underdiagnosed but the effectiveness and risks of long-term use of antidepressants in those without an appropriate indication are unknown.

Intraclass psychotropic polypharmacy was common and stable at all four time points and was associated with moderate to total dependence in performing daily activities. Thus, the most dependent people within this population receive the most psychotropics yet the impact of these changing combinations of drugs will require careful evaluation to ensure that those in receipt need all of them and benefit substantially from each of them and that their prescribing together does not expose them to unacceptable risk.

Overall, this study suggests that the use of psychotropics in this population is not optimal, that it is changing and that practices need to be further examined.

### Research implications

A surprising finding was that we could detect no effect of psychotropic use on falls or constipation in this population over the period measured. It may be that changes as a result of these drugs were most pronounced when they were initiated and are no longer obvious as interventions have been made to address these problems. An intensive evaluation of newly recruited participants aged 40 years in the Wave 4 cohort of IDS-TILDA, with regard to falls and gathering data on the fall prevention measures adopted by service providers would clarify this. Similarly, data on the diet, exercise and laxative use in those with and without constipation may help identify factors associated with the presence and/or amelioration of these problems commonly reported as associated with psychotropic use. Again, comparison of the new participants aged 40 years in the Wave 4 cohort with those from the Wave 1 cohort could give deeper insight into any changes in psychotropic patterns and the association between the two time points 10 years apart.

All of these considerations suggest that future analyses include a prospective study utilising validated clinical diagnostic instruments that examine psychotropic prescribing concurrently with access to multidisciplinary team interventions and evidence-based psychiatric service infrastructure.

### Policy implications

Although some clinical guidelines exist for appropriate prescribing for this population,^36,37^ they are not up to date, are limited in their scope, and their adoption and implementation by healthcare professionals throughout Ireland is not sufficiently promoted. The emphasis to date has been on the management of medicines by intellectual disability service providers (the HSE's National Framework for Medicines Management in Disability Services) rather than the optimisation of medicines use.^38^

Focusing on medicines management does not address the complexity of the medical needs of this vulnerable population that leads to polypharmacy, both psychotropic and non-psychotropic, which is almost ubiquitous. Policy responses should encourage changing practice to address the selection, prescribing, monitoring and evaluation of psychotropics, as other research with this population has suggested.^28^ Regular review should be required for each patient, which will inevitably identify challenges for resolving conflicting clinical priorities and for coordinating care that will then lead to change. Any required review should include all the medicines, as well as consideration of non-pharmacological alternatives and involvement of multidisciplinary teams that include access to pharmacists and those able to lead specialist non-pharmacological interventions. Only in some in-patient mental health settings are pharmacists currently utilised as part of the multidisciplinary team. In Ireland, neither mental health policy nor the intellectual disability health services envisage a role for them in optimising medicines use to meet clinical need and minimise avoidable risk, unlike the corresponding policies in the UK.

Although there has been some decrease in antipsychotic prescribing, antipsychotics remain the most frequently used psychotropic and must be a priority for action. The difficulties associated with stopping these drugs, even when they are used for behaviours, have been well documented. One approach to sustaining this decrease would be a policy-driven national campaign, similar to the STOMP (stopping over-medication of people with a learning disability, autism or both) programme in the UK, which would be a valuable first step to risk reduction for this population.

However, to formulate policies in response to the complex pattern of psychotropic use and the changes that have occurred over the study period also requires consideration of the societal and cultural context and health resourcing and infrastructure in which these medicines are prescribed. For example, there are currently no clear pathways to routine or emergency residential care for adults with disability and although there has been partial roll out of community mental health intellectual disability teams nationally there are no specialist in-patient acute psychiatric beds for adults with intellectual disability. Optimising the use of psychotropics must be an objective, but it cannot be looked at in isolation – a comprehensive approach to care provision is essential.

### Limitations

There was an absence of independent confirmation of all the self-reported medicines or conditions. However, this issue was mitigated by cross-checking the list of medicines reported in the PIQ at the time of interview to improve the accuracy of data gathered and by providing the PIQ to participants 1 week in advance of the main interview to allow them to seek support from their carers to gather information from case notes.^3^ Nevertheless, no information on reason for prescribing medicines was available. As GEE models were used, the estimates produced reflect only the changes in participant characteristics at the population level, without consideration of changes at the participant level over time. A different modelling approach would be required to reveal participant-level changes in characteristics over time and their potential effect on psychotropic drug use. Additionally, the absence of data on drug and alcohol use (addiction and dependency) meant their contributions to findings were not analysed in this study. A low incidence of substance use has been reported to date in IDS-TILDA.^11^

### Strengths

Longitudinal data from four waves of IDS-TILDA enabled us to follow the same cohort of adults with intellectual disability over a decade (2009/2010–2019/2020). Accordingly, we observed trends of psychotropic drug use, including temporal effects of its usage and factors that contributed to observed trends and extent of use.

Second, the robust nature of field researcher training and cross-checking of data resulted in 95% of participants’ medication data captured, adding to the strength of the reported results.^3^ Gathering these data as part of a large longitudinal study also enabled inclusion in analyses of associations with mental health, neurological conditions, falls and functional status.

Third, a new interviewing procedure was implemented to improve the level of accuracy for health-related conditions reporting in Wave 3 and Wave 4, allowing participants to confirm or dispute conditions reported in previous waves (Supplementary Appendix 4).

## Supporting information

Odalović et al. supplementary material Odalović et al. supplementary material

## Data Availability

The data that support the findings of this study are available from the corresponding author on reasonable request.
