# Supplementary material for: Psychotropic medicines’ prevalence, patterns and effects on cognitive and physical function in older adults with intellectual disability in Ireland: longitudinal cohort study, 2009–2020
Source: BJPsych Open. 2024 Feb 1;10(2):e39. doi: 10.1192/bjo.2023.607 (PMC10897684; doi:10.1192/bjo.2023.607)
Supplement: Odalović et al. supplementary material [file S2056472423006075sup001.docx]

Appendix 1. Flowchart for the study





Appendix 2. Health outcomes defining and questioning procedure, in the original scale and the modifications undertaken

| **Health Outcome** | Questioning in the original scale and the modifications undertaken |
| --- | --- |
| **Functional status** assessed by the Barthel Index (BI) | The BI is a measure of assessing disability in those receiving rehabilitation for neuromuscular and musculoskeletal conditions and has become a reliable method of measuring function in older populations. It consists of an ordinal scale of 10 activities of daily living and considers the level of dependence an individual has in mobility, using stairs, dressing, bathing, grooming, feeding, transfer, toileting, and bladder and bowel continence. A modified form of BI activities of daily living was created for this population *(Appendix 3).* Each participant was given a composite score between 0 and 20 based on their self-/proxy-report of difficulty experienced with each activity. The BI scores were categorized as total dependence (0–4), severe dependence (5–12), moderate dependence (13–18), mild dependence (19) and total independence (20) (17). Participants with two or more missing values (i.e. those who answered “don’t know”, gave an unclear response or preferred not to answer) were excluded from the BI evaluation as this method has been used by team members previously (11). |
| **Chronic constipation** (yes/no) | Participants/proxies were asked “Have you ever had a doctor’s diagnosis of chronic constipation?” in Wave 1 and Wave 2, with answers “yes”, “no” or “don’t know” (the answer “don’t know” was considered as missing data). To confirm the accuracy of already provided information in previous wave, participants had the opportunity in Wave 3 and Wave 4 to confirm or dispute a previously recorded answer, as well as to report the condition if it is present (*Appendix 4*). This interviewing procedure was implemented in questioning of all health conditions. The disputed answers were only used in prevalence calculation in the corresponding wave, while the prevalence already published for the previous wave was not changed. |
| **Falls in the past year** (yes/no) | At each wave, participants/proxies were asked “In the past year have you had any fall including a slip or trip in which you lost your balance and landed on the floor or ground or lower level?” to which they answered “yes”, “no” or “don’t know”. |
| **Dementia** (yes/no) | In Wave 1, the presence of dementia was calculated by combining responses to the following questions: “Has a doctor ever told you that you have Alzheimer’s disease?”, “Has the doctor ever told you that you have dementia, organic brain syndrome or senility?” and “Has a doctor ever told you that you have serious memory impairment?”; as well as the reporting of any anti-dementia drug (identified using the ATC code N06D). In Wave 2, dementia was calculated using the questions “Has a doctor ever told you that you have Alzheimer’s disease?” and “Has a doctor ever told you that you have dementia, organic brain syndrome or senility?” as well as the reporting of any anti-dementia drug. To survey the prevalence of dementia or Alzheimer’s disease in Wave 3 and Wave 4, the procedure presented in *Appendix 4* is implemented and the prevalence was calculated alongside the reporting of any anti-dementia drug. |
| **Ability to walk 100 yards** | Participants/proxies were asked “Please indicate the level of difficulty, if any, you have with walking 100 yards”. Answers were combined in two categories: “no/some difficulty” and “a lot of difficulty/cannot do at all”. |
| **Epilepsy** (yes/no) | Participants/proxies were asked “Have you ever had a doctor’s diagnosis of epilepsy?” in Wave 1 and Wave 2, with possible answers “yes”, “no” or “don’t know”. *Appendix 4* presents the questioning procedure in Wave 3 and Wave 4. |
| **Mental health condition** (yes/no) | Participants/proxies were asked “Has a doctor ever told you that you have an emotional, nervous or psychiatric condition?” in Wave 1 and Wave 2, with possible answers “yes”, “no” or “don’t know”. *Appendix 4* presents the questioning procedure in Wave 3 and Wave 4. |

Appendix 3. Questioning methodology with regards to Barthel index

|  | Wave 1 | Wave 2 | Wave 3 | Wave 4 |
| --- | --- | --- | --- | --- |
| Mobility | Please indicate the level of difficulty, if any, you have with walking 100 yards | Please indicate the level of difficulty, if any, you have with walking 100 yards | Please indicate the level of difficulty, if any, you have with walking 100 yards | Please indicate the level of difficulty, if any, you have with walking 100 yards |
|  | No difficulty, some difficulty, a lot of difficulty, cannot do at all | No difficulty, some difficulty, a lot of difficulty, cannot do at all | No difficulty, some difficulty, a lot of difficulty, cannot do at all | No difficulty, some difficulty, a lot of difficulty, cannot do at all |
| Stairs | Please indicate the level of difficulty, if any, you have with climbing one flight of stairs without resting. | Please indicate the level of difficulty, if any, you have with climbing one flight of stairs without resting. | Please indicate the level of difficulty, if any, you have with climbing one flight of stairs without resting. | Please indicate the level of difficulty, if any, you have with climbing one flight of stairs without resting. |
|  | No difficulty, some difficulty, a lot of difficulty, cannot do at all | No difficulty, some difficulty, a lot of difficulty, cannot do at all | No difficulty, some difficulty, a lot of difficulty, cannot do at all | No difficulty, some difficulty, a lot of difficulty, cannot do at all |
| Dressing | Please indicate the level of difficulty, if any, you have with dressing, including putting on shoes and socks. | Please indicate the level of difficulty, if any, you have with dressing, including putting on shoes and socks. | Please indicate the level of difficulty, if any, you have with dressing, including putting on shoes and socks. | Please indicate the level of difficulty, if any, you have with dressing. |
|  | No difficulty, some difficulty, a lot of difficulty, cannot do at all | No difficulty, some difficulty, a lot of difficulty, cannot do at all | No difficulty, some difficulty, a lot of difficulty, cannot do at all | No difficulty, some difficulty, a lot of difficulty, cannot do at all |
| Bathing | Please indicate the level of difficulty, if any, you have with bathing or showering | Please indicate the level of difficulty, if any, you have with bathing or showering | Please indicate the level of difficulty, if any, you have with bathing or showering | Please indicate the level of difficulty, if any, you have with bathing or showering |
|  | No difficulty, some difficulty, a lot of difficulty, cannot do at all | No difficulty, some difficulty, a lot of difficulty, cannot do at all | No difficulty, some difficulty, a lot of difficulty, cannot do at all | No difficulty, some difficulty, a lot of difficulty, cannot do at all |
| Grooming | Please indicate the level of difficulty, if any, you have with cleaning your teeth/taking care of your dentures | Please indicate the level of difficulty, if any, you have with cleaning your teeth/taking care of your dentures | What best describes the physical assistance you get from someone else clean your teeth? | What best describes the physical help you get from someone else to clean your teeth? |
|  | No difficulty, some difficulty, a lot of difficulty, cannot do at all | No difficulty, some difficulty, a lot of difficulty, cannot do at all | Without assistance, with assistance, totally dependent on another person, do not clean teeth, do not have teeth to clean | Without help, with a little help, with a lot of help, do not clean teeth, no teeth to clean |
| Feeding | Please indicate the level of difficulty, if any, you have with eating such as cutting up your food, use of utensils, drinking from a cup/glass etc? | Please indicate the level of difficulty, if any, you have with eating such as cutting up your food, use of utensils, drinking from a cup/glass etc? | Please indicate the level of difficulty, if any, you have with eating such as cutting up your food, use of utensils, drinking from a cup/glass etc? | Please indicate the level of difficulty, if any, you have with eating. |
|  | No difficulty, some difficulty, a lot of difficulty, cannot do at all | No difficulty, some difficulty, a lot of difficulty, cannot do at all | No difficulty, some difficulty, a lot of difficulty, cannot do at all | No difficulty, some difficulty, a lot of difficulty, cannot do at all |
| Transfer | Please indicate the level of difficulty, if any, you have with getting in or out of bed. | Please indicate the level of difficulty, if any, you have with getting in or out of bed. | Please indicate the level of difficulty, if any, you have with getting in or out of bed. | Please indicate the level of difficulty, if any, you have with: bed – in and out. |
|  | No difficulty, some difficulty, a lot of difficulty, cannot do at all | No difficulty, some difficulty, a lot of difficulty, cannot do at all | No difficulty, some difficulty, a lot of difficulty, cannot do at all | No difficulty, some difficulty, a lot of difficulty, cannot do at all |
| Toileting | Please indicate the level of difficulty, if any, you have with using the toilet, including getting up or down. | Please indicate the level of difficulty, if any, you have with using the toilet, including getting up or down. | Please indicate the level of difficulty, if any, you have with using the toilet, including getting up or down. | Please indicate the level of difficulty, if any, you have with using the toilet. |
|  | No difficulty, some difficulty, a lot of difficulty, cannot do at all | No difficulty, some difficulty, a lot of difficulty, cannot do at all | No difficulty, some difficulty, a lot of difficulty, cannot do at all | No difficulty, some difficulty, a lot of difficulty, cannot do at all |
| Bladder continence | During the last 12 months, have you lost any amount of urine beyond your control? Did this happen more than once during a 1- month period? | During the last 12 months, have you lost any amount of urine beyond your control? Did this happen more than once during a 1- month period? | During the last 12 months, have you lost any amount of urine beyond your control? Did this happen more than once during a 1- month period? | During the last 12 months, have you lost any amount of urine beyond your control? Did this happen more than once during a 1- month period? |
|  | Yes & yes, yes & no, no | Yes & yes, yes & no, no | Yes & yes, yes & no, no | Yes & yes, yes & no, no |
| Bowel continence | During the last 12 months, have you lost any amount of faeces beyond your control? Did this happen more than once during a 1- month period? | During the last 12 months, have you lost any amount of faeces beyond your control? Did this happen more than once during a 1- month period? | During the last 12 months, have you lost any amount of faeces beyond your control? Did this happen more than once during a 1- month period? | During the last 12 months, have you lost any amount of faeces beyond your control? Did this happen more than once during a 1- month period? |
|  | Yes & yes, yes & no, no | Yes & yes, yes & no, no | Yes & yes, yes & no, no | Yes & yes, yes & no, no |

Appendix 4. Questioning procedure for health conditions in Wave 3 and Wave 4.





Appendix 5. Comparison of mental health conditions prevalence between four time points in 10-year period of time

| **Mental health condition** | **Wave 1 (2009/10),**  **N = 433, n, %** | **Wave 2 (2013/14),**  **N = 433, n, %** | **Wave 3 (2016/17),**  **N = 433, n, %** | **Wave 4 (2019/20),**  **N = 433, n, %** | **Difference between time points, p value** |
| --- | --- | --- | --- | --- | --- |
| **Psychosis** |  |  |  |  | >0.05 |
| Yes | 28 (6.7) | 18 (4.3) | 18 (4.2) | 14 (3.3) |  |
| No | 387 (93.3) | 396 (95.7) | 414 (95.8) | 412 (96.7) |  |
| Missing | n = 18 | n =19 | n = 1 | n = 7 |  |
| **Depression** |  |  |  |  | <0.05, Wave 1 vs. Wave4 |
| Yes | 87 (21.0) | 78 (18.8) | 72 (16.7) | 52 (12.2) |  |
| No | 328 (79.0) | 336 (81.2) | 360 (83.3) | 374 (87.8) |  |
| Missing | n = 18 | n =19 | n = 1 | n = 7 |  |
| **Manic depression** |  |  |  |  | >0.05 |
| Yes | 15 (3.6) | 7 (1.7) | 11 (2.5) | 13 (3.1) |  |
| No | 400 (96.4) | 407 (98.3) | 421 (97.5) | 413 (96.9) |  |
| Missing | n = 18 | n =19 | n = 1 | n = 7 |  |
| **Anxiety** |  |  |  |  | >0.05 |
| Yes | 126 (30.4) | 125 (30.1) | 147 (34.0) | 131 (30.8) |  |
| No | 289 (69.6) | 290 (69.9) | 285 (66.0) | 295 (69.2) |  |
| Missing | n = 18 | n =18 | n = 1 | n = 7 |  |
| **Schizophrenia** |  |  |  |  |  |
| Yes | 20 (4.8) | 20 (4.8) | 18 (4.2) | 12 (2.8) | >0.05 |
| No | 394 (95.2) | 394 (95.2) | 414 (95.8) | 414 (97.2) |  |
| Missing | n = 19 | n =19 | n = 1 | n = 7 |  |
| **Hallucinations** |  |  |  |  | >0.05 |
| Yes | 17 (4.1) | 11 (2.7) | 10 (2.3) | 15 (3.5) |  |
| No | 399 (95.9) | 403 (97.3) | 422 (97.7) | 411 (96.5) |  |
| Missing | n = 17 | n =19 | n = 1 | n = 7 |  |
| **Emotional problems** |  |  |  |  | p<0.05, Wave 2 vs. Wave 4 |
| Yes | 63 (15.1) | 46 (11.1) | 66 (15.3) | 77 (18.1) |  |
| No | 353 (84.9) | 369 (88.9) | 366 (84.7) | 349 (81.9) |  |
| Missing | n = 17 | n =18 | n = 1 | n = 7 |  |
| **Mood swings** |  |  |  |  | >0.05 |
| Yes | 92 (22.2) | 78 (18.8) | 73 (16.9) | 82 (19.2) |  |
| No | 323 (77.8) | 337 (81.2) | 359 (83.1) | 344 (80.8) |  |
| Missing | n = 18 | n =18 | n = 1 | n = 7 |  |

Note: the waves between the significance in frequencies is significantly different are noted within last column of the table

Appendix 6. Psychotropic drug use over 10-year period (N=433)

| **Drug category** | **Wave 1: 2009/10, n, %, 95% CI, N=433** | **Wave 2: 2013/14, n, %, 95% CI, N=433** | **Wave 3: 2016/17, n, %, 95% CI, N=433** | **Wave 4: 2019/20 n, %,95% CI, N=433** | **Difference in the proportions between waves (p value) *** |
| --- | --- | --- | --- | --- | --- |
| **Any psychotropic** |  |  |  |  |  |
| No | 165, 38.1 (33.5-42.9) | 155, 35.8 (31.3-40.5) | 168, 38.8 (34.2-43.6) | 162, 37.4 (32.8-42.2) | p>0.05 |
| Yes | 268, 61.9 (57.1-66.5) | 278, 64.2 (59.5-68.7) | 265, 61.2 (56.4-65.8) | 271, 62.6 (57.8-67.2) |  |
| **Antipsychotics** |  |  |  |  |  |
| No | 228, 52.7 (47.8-57.4) | 227, 52.4 (47.6-57.2) | 236, 54.5 (49.7-59.3) | 240, 55.4 (50.6-60.2) | p>0.05 |
| Yes | 205, 47.3 (42.6-52.2) | 206, 47.6 (42.8-52.4) | 197, 45.5 (40.7-50.3) | 193, 44.6 (39.8-49.4) |  |
| **Anxiolytics** |  |  |  |  |  |
| No | 323, 74.6 (70.2-78.6) | 317, 73.2 (68.8-77.3) | 356, 82.2 (78.3-85.7) | 357, 82.4 (78.5-85.9) | **<0.05**  Wave 1 vs. Wave 3  Wave 1 vs. Wave 4  **<0.001**  Wave 2 vs. Wave 3  Wave 2 vs. Wave 4 |
| Yes | 110, 25.4 (21.4-29.8) | 116, 26.8 (22.7-31.2) | 77, 17.8 (14.3-21.7) | 76, 17.6 (14.1-21.5) |  |
| **Hypnotics/Sedatives** |  |  |  |  |  |
| No | 372, 85.9 (82.3-89.1) | 374, 86.4 (82.8-89.5) | 393, 90.8 (87.6-93.3) | 394, 91.0 (87.9-93.5) | **<0.05**  Wave 1 vs. Wave 3  Wave 1 vs. Wave 4  Wave 2 vs. Wave 3  Wave 2 vs. Wave 4 |
| Yes | 61, 14.1 (11.0-17.8) | 59, 13.6 (10.5-17.2) | 40, 9.2 (6.7-12.4) | 39, 9.0 (6.5-12.1) |  |
| **Mood stabilising agents** |  |  |  |  | **p<0.05**  Wave 3 vs. Wave 4  Wave 2 vs. Wave 4  **p<0.001**  Wave 1 vs. Wave 4 |
| No | 383, 88.5 (85.1-91.3) | 378, 87.3 (83.8-90.3) | 375, 86.6 (83.0-89.7) | 370, 85.5 (81.8-88.6) |  |
| Yes | 50, 11.5 (8.7-14.9) | 55, 12.7 (9.7-16.2) | 58, 13.4 (10.3-17.0) | 63, 14.6 (11.4-18.2) |  |
| **Antidepressants** |  |  |  |  |  |
| No | 309, 71.4 (66.9-75.6) | 298, 68.8 (64.2-73.2) | 286, 66.1 (61.4-70.5) | 278, 64.2 (59.5-68.7) | **p<0.05**  Wave 1 vs. Wave 3  Wave 2 vs. Wave 4  **p<0.001**  Wave 1 vs. Wave 4 |
| Yes | 124, 28.6 (24.4-33.2) | 135, 31.2 (26.8-35.8) | 147, 34.0 (29.5-38.6) | 155, 35.8 (31.3-40.5) |  |

Abbreviation: CI-confidence interval, *specific waves which significantly differed from each other in pairwise comparisons were presented in the table

Appendix 7. Individual drug prevalence change across the waves, 2009/10-2019/20

| **INN** | **Wave 1: 2009/10, n, %, N=433** | **Wave 2: 2013/14, n, %, N=433** | **Wave 3: 2016/17, n, %, N=433** | **Wave 4: 2019/20, 2019/20 n, %, N=433** | **Difference in the proportions between waves (p value) *** |
| --- | --- | --- | --- | --- | --- |
| **Risperidone, N05AX08** |  |  |  |  | >0.05 |
| No | 355, 82.0 | 353, 81.5 | 366, 84.5 | 365, 84.3 |  |
| Yes | 78, 18.0 | 80, 18.5 | 67, 15.5 | 68, 15.7 |  |
| **Olanzapine, N05AH03** |  |  |  |  | >0.05 |
| No | 370, 85.5 | 370, 85.5 | 370, 85.5 | 370, 85.5 |  |
| Yes | 63, 14.5 | 63, 14.5 | 63, 14.5 | 63, 14.5 |  |
| **Diazepam, N05BA01** |  |  |  |  | **<0.001**  Wave 1 vs. Wave 3  Wave 1 vs. Wave 4  Wave 2 vs. Wave 4  **<0.05**  Wave 2 vs. Wave 3 |
| No | 365, 84.3 | 376, 86.8 | 402, 92.8 | 405, 93.5 |  |
| Yes | 68, 15.7 | 57, 13.2 | 31, 7.2 | 28, 6.5 |  |
| **Chlorpromazine, N05AA01** |  |  |  |  | **<0.001**  Wave 1 vs. Wave 4  **<0.05**  Wave 1 vs. Wave 3  Wave 2 vs. Wave 4 |
| No | 387, 89.4 | 393, 90.8 | 403, 91.1 | 406, 93.8 |  |
| Yes | 46, 10.6 | 40, 9.2 | 30, 6.9 | 27, 6.2 |  |
| **Lorazepam, N05BA06** |  |  |  |  | **<0.001**  Wave 1 vs. Wave 4  **<0.05**  Wave 1 vs. Wave 3  Wave 2 vs. Wave 3  Wave 2 vs. Wave 4 |
| No | 392, 91.2 | 395, 91.2 | 413, 95.4 | 417, 96.3 |  |
| Yes | 41, 9.5 | 38, 8.8 | 20, 4.6 | 16, 3.7 |  |
| **Haloperidol, N05AD01** |  |  |  |  | >0.05 |
| No | 407, 94.0 | 409, 94.5 | 410, 94.7 | 411, 94.9 |  |
| Yes | 26, 6.0 | 24, 5.5 | 23, 5.3 | 22, 5.1 |  |
| **Zopiclone, N05CF01** |  |  |  |  | >0.05 |
| No | 414, 95.6 | 416, 96.1 | 422, 97.5 | 419, 96.8 |  |
| Yes | 19, 4.4 | 17, 3.9 | 11, 2.5 | 14, 3.2 |  |
| **Carbamazepine, N03AF01** |  |  |  |  | >0.05 |
| No | 347, 80.1 | 347, 80.1 | 353, 81.5 | 358, 82.7 |  |
| Yes | 86, 19.9 | 86, 19.9 | 80, 18.5 | 75, 17.3 |  |
| **Escitalopram, N06AB10** |  |  |  |  | >0.05 |
| No | 411, 94.9 | 406, 93.8 | 404, 93.3 | 407, 94.0 |  |
| Yes | 22, 5.1 | 27, 6.2 | 29, 6.7 | 26, 6.0 |  |
| **Valproic Acid, N03AG01** |  |  |  |  | >0.05 |
| No | 356, 82.2 | 351, 81.1 | 349, 80.6 | 343, 79.2 |  |
| Yes | 77, 17.8 | 82, 18.9 | 84, 19.4 | 90, 20.8 |  |
| **Quetiapine, N05AH04** |  |  |  |  | **<0.05**  Wave 1 vs. Wave 4 **<0.05**  Wave 1 vs. Wave 3 |
| No | 423, 97.7 | 415, 95.8 | 412, 95.2 | 408, 94.2 |  |
| Yes | 10, 2.3 | 18, 4.2 | 21, 4.8 | 25, 5.8 |  |
| **Paroxetine, N06AB05** |  |  |  |  | >0.05 |
| No | 414, 95.6 | 416, 96.1 | 417, 96.1 | 418, 96.5 |  |
| Yes | 19, 4.4 | 17, 3.9 | 16, 3.7 | 15, 3.5 |  |
| **Citalopram, N06AB04** |  |  |  |  | >0.05 |
| No | 420, 97.0 | 420, 97.0 | 421, 97.2 | 424, 97.9 |  |
| Yes | 13, 3.0 | 13, 3.0 | 12, 2.8 | 9, 2.1 |  |
| **Fluoxetine, N06AB03** |  |  |  |  | >0.05 |
| No | 416, 96.1 | 416, 96.1 | 412, 95.2 | 413, 95.4 |  |
| Yes | 17, 3.9 | 17, 3.9 | 21, 4.8 | 20, 4.6 |  |
| **Sertraline, N06AB06** |  |  |  |  | **<0.001**  Wave 1 vs. Wave 4  Wave 2 vs. Wave 4  **<0.05**  Wave 1 vs. Wave 3  Wave 3 vs. Wave 4 |
| No | 420, 97.0 | 412, 95.2 | 407, 94.0 | 390, 90.1 |  |
| Yes | 13, 3.0 | 21, 4.8 | 26, 6.0 | 43, 9.9 |  |
| **Alprazolam, N05BA12** |  |  |  |  | >0.05 |
| No | 418, 96.5 | 421, 97.2 | 420, 97.0 | 420, 97.0 |  |
| Yes | 15, 3.5 | 12, 2.8 | 13, 3.0 | 13, 3.0 |  |
| **Zolpidem, N05CF02** |  |  |  |  | >0.05 |
| No | 423, 97.7 | 422, 97.5 | 424, 97.9 | 423, 97.7 |  |
| Yes | 10, 2.3 | 11, 2.5 | 9, 2.1 | 10, 2.3 |  |

*specific waves which significantly differed from each other in pairwise comparisons were presented in the table

Appendix 8. Intraclass psychotropic polypharmacy prevalence change across the waves, 2009/10-2019/20

|  | **Wave 1: 2009/10, n, %, 95% CI, N=433** | **Wave 2: 2013/14, n, %, 95% CI, N=433** | **Wave 3: 2016/17, n, %, 95% CI, N=433** | **Wave 4: 2019/20, 2019/20 n, %, 95% CI, N=433** | **Difference in the proportions between waves for two categories (use of one / use of two or more psychotropics)** **(p value) *** |
| --- | --- | --- | --- | --- | --- |
| **Psychotropic polypharmacy** |  |  |  |  | p>0.05 |
| **0** | 165, 38.1 (33.5-42.9) | 155, 35.8 (31.3-40.5) | 168, 38.8 (34.2-43.6) | 162, 37.4 (32.8-42.2) |  |
| **1** | 89, 20.6 (16.8-24.7) | 93, 21.5 (17.7-25.7) | 94, 21.7 (17.9-25.9) | 105, 24.2 (20.3-28.6) |  |
| **2+** | 179, 41.3 (36.7-46.1) | 185, 42.7 (38.0-47.5) | 171, 39.5 (34.9-44.3) | 166, 38.3 (33.7-43.1) |  |
| **Median** | 2 | 2 | 2 | 2 |  |
| **IQR** | 6 | 5 | 6 | 7 |  |
| **Minimum** | 1 | 1 | 1 | 1 |  |
| **Maximum** | 7 | 6 | 7 | 8 |  |
| **1^st^ QT** | 1 | 1 | 1 | 1 |  |
| **2^nd^ QT** | 2 | 2 | 2 | 2 |  |
| **3^rd^ QT** | 3 | 3 | 3 | 3 |  |
| **Antipsychotic intraclass polypharmacy** |  |  |  |  | >0.05 |
| **0** | 228, 52.7 (47.8-57.4) | 227, 52.4 (47.6-57.2) | 236, 54.5 (49.7-59.3) | 240, 55.4 (50.6-60.2) |  |
| **1** | 151, 34.9 (30.4-39.6) | 161, 37.2 (32.6-41.9) | 154, 35.6 (31.1-40.3) | 151, 34.9 (30.4-39.6) |  |
| **2+** | 54, 12.5 (9.5-16.0) | 45, 10.4 (7.7-13.7) | 43, 9.9 (7.3-13.1) | 42, 9.7 (7.1-12.9) |  |
| **Median** | 1 | 1 | 1 | 1 |  |
| **IQR** | 2 | 2 | 2 | 2 |  |
| **Minimum** | 1 | 1 | 1 | 1 |  |
| **Maximum** | 3 | 3 | 3 | 3 |  |
| **1^st^ QT** | 1 | 1 | 1 | 1 |  |
| **2^nd^ QT** | 1 | 1 | 1 | 1 |  |
| **3^rd^ QT** | 2 | 1 | 1 | 1 |  |
| **Anxiolytic intraclass polypharmacy** |  |  |  |  | >0.05 |
| **0** | 323, 74.6 (70.2-78.6) | 317, 73.2 (68.8-77.3) | 356, 82.2 (78.3-85.7) | 357, 82.4 (78.5-85.9) |  |
| **1** | 102, 23.6 (19.6-27.8) | 105, 24.2 (20.3-28.6) | 68, 15.7 (12.4-19.5) | 71, 16.4 (13.0-20.2) |  |
| **2+** | 8, 1.8 (0.8-3.6) | 11, 2.5 (1.3-4.5) | 9, 2.1 (1.0-3.9) | 5, 1.2 (0.4-2.7) |  |
| **Median** | 1 | 1 | 1 | 1 |  |
| **IQR** | 1 | 2 | 1 | 1 |  |
| **Minimum** | 1 | 1 | 1 | 1 |  |
| **Maximum** | 2 | 3 | 2 | 2 |  |
| **1^st^ QT** | 1 | 1 | 1 | 1 |  |
| **2^nd^ QT** | 1 | 1 | 1 | 1 |  |
| **3^rd^ QT** | 1 | 1 | 1 | 1 |  |
| **Hypnotics/Sedatives intraclass polypharmacy** |  |  |  |  | >0.05 |
| **0** | 372, 85.9 (82.3-89.1) | 374, 86.4 (82.8-89.5) | 393, 90.8 (87.6-93.3) | 394, 91.0 (87.9-93.5) |  |
| **1** | 61, 14.1 (11.0-17.7) | 58, 13.4 (10.3-17.0) | 37, 8.5 (6.1-11.6) | 35, 8.1 (5.7-11.1) |  |
| **2+** | / | 1, 0.2 (0.01-1.3) | 3, 0.7 (0.1-2.0) | 4, 0.9 (0.3-2.4) |  |
| **Median** | 1 | 1 | 1 | 1 |  |
| **IQR** | 0 | 1 | 1 | 1 |  |
| **Minimum** | 1 | 1 | 1 | 1 |  |
| **Maximum** | 1 | 2 | 2 | 2 |  |
| **1^st^ QT** | 1 | 1 | 1 | 1 |  |
| **2^nd^ QT** | 1 | 1 | 1 | 1 |  |
| **3^rd^ QT** | 1 | 1 | 1 | 1 |  |
| **Mood stabilising agents intraclass polypharmacy** |  |  |  |  | p>0.05 |
| **0** | 383, 88.5 (85.1-91.3) | 378, 87.3 (83.8-90.3) | 375, 86.6 (83.0-89.7) | 370, 85.5 (81.8-88.6) |  |
| **1** | 38, 8.8 (6.3-11.9) | 45, 10.4 (7.7-13.7) | 47, 10.9 (8.1-14.2) | 49, 11.3 (8.5-14.7) |  |
| **2+** | 12, 2.8 (1.4-4.8) | 10, 2.3 (1.1-4.2) | 11, 2.5 (1.3-4.5) | 14, 3.2 (1.8-5.4) |  |
| **Median** | 1 | 1 | 1 | 1 |  |
| **IQR** | 2 | 2 | 2 | 2 |  |
| **Minimum** | 1 | 1 | 1 | 1 |  |
| **Maximum** | 3 | 3 | 3 | 3 |  |
| **1^st^ QT** | 1 | 1 | 1 | 1 |  |
| **2^nd^ QT** | 1 | 1 | 1 | 1 |  |
| **3^rd^ QT** | 1.25 | 1 | 1 | 1 |  |
| **Antidepressants intraclass polypharmacy** |  |  |  |  | >0.05 |
| **0** | 309, 71.4 (66.9-75.6) | 298, 68.8 (64.2-73.2) | 286, 66.1 (61.4-70.5) | 278, 64.2 (59.5-68.7) |  |
| **1** | 120, 27.7 (23.6-32.2) | 130, 30.0 (25.7-34.6) | 139, 32.1 (27.7-36.7) | 148, 34.2 (29.7-38.7) |  |
| **2+** | 4, 0.9 (0.3-2.4) | 5, 1.2 (0.4-2.7) | 8, 1.8 (0.8-3.6) | 7, 1.6 (0.7-3.3) |  |
| **Median** | 1 | 1 | 1 | 1 |  |
| **IQR** | 1 | 1 | 1 | 1 |  |
| **Minimum** | 1 | 1 | 1 | 1 |  |
| **Maximum** | 2 | 2 | 2 | 2 |  |
| **1^st^ QT** | 1 | 1 | 1 | 1 |  |
| **2^nd^ QT** | 1 | 1 | 1 | 1 |  |
| **3^rd^ QT** | 1 | 1 | 1 | 1 |  |

Abbreviation: CI-confidence interval

Appendix 9. Interclass psychotropic polypharmacy prevalence change across the waves, 2009/10-2019/20

| **Psychotropic subcategories** | **Wave 1: 2009/10, n, %, 95% CI, N=433** | **Wave 2: 2013/14, n, %, 95% CI, N=433** | **Wave 3: 2016/17, n, %, N=433** | **Wave 4: 2019/20, n, %, 95% CI, N=433** | **Difference in the proportions between waves (p value) *** |
| --- | --- | --- | --- | --- | --- |
| **Antipsychotic, antidepressant** |  |  |  |  | p>0.05 |
| No | 343, 79.2 (75.1-82.9) | 339, 78.3 (74.1-82.1) | 334, 77.1 (72.9-81.0) | 335, 77.4 (73.1-81.2) |  |
| Yes | 90, 20.8 (17.1-24.9) | 94, 21.7 (17.9-25.9) | 99, 22.9 (19.0-27.1) | 98, 22.6 (18.8-26.9) |  |
| **Antipsychotic, anxiolytic, sedative** |  |  |  |  | **p<0.05**  Wave 2 vs. Wave 3  **p<0.001**  Wave 1 vs. Wave 4  Wave 2 vs. Wave 4 |
| No | 406, 93.8 (91.1-95.9) | 400, 92.4 (89.5-94.7) | 418, 96.5 (94.4-98.1) | 429, 99.1 (97.7-99.8) |  |
| Yes | 27, 6.2 (4.2-8.9) | 33, 7.6 (5.3-10.5) | 15, 3.5 (2.0-5.7) | 4, 0.9 (0.3-2.4) |  |
| **Antipsychotic, anxiolytics, sedative, antidepressant** |  |  |  |  | **p<0.05**  Wave 2 vs. Wave 4  **p< 0.001**  Wave 1 vs. Wave 4 |
| No | 415, 95.8 (93.5-97.5) | 417, 96.3 (94.1-97.9) | 423, 97.7 (95.8-98.9) | 431, 99.5 (98.3-99.9) |  |
| Yes | 18, 4.2 (2.5-6.5) | 16, 3.7 (2.1-5.9) | 10, 2.3 (1.1-4.2) | 2, 0.5 (0.06-1.7) |  |

*specific waves which significantly differed from each other in pairwise comparisons were presented in the table; Abbreviation: CI-confidence interval

Appendix 10. Factors associated with higher number of psychotropic drugs use (N=433)

| **Characteristic** | **Any psychotropic estimates** | **Antipsychotics estimates** | **Anxiolytics, OR (CI)** | **Hypnotics/ Sedatives, OR (CI)** | **Mood-stabilising agents, OR (CI)** | **Antidepressants, OR (CI)** |
| --- | --- | --- | --- | --- | --- | --- |
| **Age (years)** |  |  |  |  |  |  |
| 40-49 | 1 | 1 | 1 | 1 | 1 | 1 |
| 50-64 | 0.96 (0.86-1.06) | 1.05 (0.86-1.29) | 0.83 (0.66-1.05) | 1.00 (0.62-1.61) | 0.82 (0.59-1.15) | 0.04 (0.88-1.22) |
| 65+ | 0.92 (0.79-1.08) | 0.98 (0.87-1.10) | 0.71 (0.49-1.03) | 0.88 (0.66-1.19) | 0.92 (0.56-1.51) | 0.88 (0.69-1.11) |
| **Gender** |  |  |  |  |  |  |
| Male | 1 | 1 | 1 | 1 | 1 | 1 |
| Female | 1.00 (0.85-1.18) | **0.79 (0.65-0.96)** | 0.87 (0.67-1.13) | 1.15 (0.74-1.80) | 0.96 (0.54-1.73) | 1.42 (1.09-1.85) |
| **Residence** |  |  |  |  |  |  |
| Independent/Family | 1 | 1 | 1 | 1 | 1 | 1 |
| Community group home | 1.33 (0.98-1.80) | 1.34 (0.97-1.86) | **2.10 (1.07-4.13)** | 1.28 (0.74-2.21) | 1.12 (0.50-2.49) | 1.40 (0.97-2.01) |
| Residential care | **1.46 (1.09-1.96)** | **1.40 (1.02-1.92)** | **2.32 (1.17-4.59)** | 1.34 (0.77-2.33) | **1.75 (0.83-3.71)** | **1.48 (1.02-2.13)** |
| **ID level** |  |  |  |  |  |  |
| Mild | 1 | 1 | 1 | 1 | 1 | 1 |
| Moderate | 1.17 (0.93-1.48) | 1.02 (0.77-1.34) | **1.60 (1.02-2.52)** | 1.96 (0.96-4.02) | 2.30 (0.76-6.99) | 0.86 (0.66-1.11) |
| Severe/Profound | **1.29 (1.02-1.65)** | 1.16 (0.87-1.57) | 1.33 (0.84-2.12) | **2.30 (1.11-4.78)** | 2.12 (0.60-7.52) | **0.67 (0.48-0.93)** |
| **Epilepsy** |  |  |  |  |  |  |
| No | 1 | 1 | 1 | 1 | 1 | 1 |
| Yes | **0.68 (0.56-0.81)** | 0.85 (0.69-1.05) | **1.40 (1.09-1.80)** | 1.40 (0.98-2.02) | **0.05 (0.01-0.47)** | **0.77 (0.63-0.96)** |
| **Mental health condition** |  |  |  |  |  |  |
| No | 1 | 1 | 1 | 1 | 1 | 1 |
| Yes | **1.65 (1.44-1.89)** | **1.78 (1.50-2.11)** | **2.17 (1.66-2.84)** | **1.74 (1.23-2.45)** | **2.27 (1.34-3.85)** | **1.67 (1.38-2.02)** |
| **Time** |  |  |  |  |  |  |
| Wave 1 (2019/10) | 1 | 1 | 1 | 1 | 1 | 1 |
| Wave 2 (2013/14) | 1.03 (0.96-1.11) | 0.97 (0.89-1.05) | 1.12 (0.92-1.37) | 1.05 (0.83-1.32) | 1.18 (0.93-1.51) | 1.07 (0.95-1.20) |
| Wave 3 (2016/17) | 0.97 (0.88-1.06) | 0.91 (0.81-1.01) | 0.75 (0.57-0.98) | 0.72 (0.52-1.01) | 1.46 (1.16-1.85) | **1.20 (1.06-1.36)** |
| Wave 4 (2019/20) | 0.93 (0.84-1.03) | 0.90 (0.79-1.01) | 0.76 (0.57-1.01) | 0.75 (0.51-1.09) | 1.53 (1.13-2.08) | **1.28 (1.10-1.49)** |

Bold: p<0.05

Appendix 11. Studies on psychotropic use comparisons

| **Study** | **Period of observation** | **Population** | **Any psychotropic** | **Antipsychotics** | **Anxiolytics** | **Benzodiazepines** | **Hypnotics/ Sedatives** | **Hypnotics/ Anxiolytics** | **Mood-stabilising agents** | **Antidepressants** | **Rank order of the most frequent psychotropic medicines usage in the first time point** | **Rank order of the most frequent psychotropic medicines usage in the last time point** |
| --- | --- | --- | --- | --- | --- | --- | --- | --- | --- | --- | --- | --- |
| Present study (2023) | 2009/10-2019/20 | 433 participants with ID, ≥40 years of age | 61.9-62.6 %, p>0.05 | 47.3-44.6%, p>0.05 | 25.4-17.6%, <0.05 | / | 14.1-9.0%,  <0.05 | / | 11.5-14.6%,  p<0.001 | 28.6-35.8%, p<0.001 | 1. Antipsychotics  2. Antidepressants  3. Anxiolytics  4. Hypnotics/ Sedatives  5. Mood-stabilising agents | 1. Antipsychotics  2. Antidepressants  3. Anxiolytics  4. Mood-stabilising agents  5. Hypnotics/ Sedatives |
| Psychotropic medication use in adults with intellectual disability in Queensland, Australia, from 1999 to 2015: a cohort study, Song et al. (2020) (18) | 1999/01-2012/15 | Participants with ID, ≥18 years of age  - 138 participants at the baseline  -92 participants at the last time point | 43.3-54.2% | 26.7-27.7% | 4.4-8.4% | / | 3.3-1.2% | / | / | 16.7-36.1% | 1. Antipsychotics  2. Antidepressants  3. Anxiolytics  4. Hypnotics/ Sedatives | 1. Antidepressants  2. Antipsychotics  3. Anxiolytics  4. Hypnotics/ Sedatives |
| Changes over a decade in psychotropic prescribing for people with intellectual disabilities: prospective cohort study, Henderson et al. (2020) (19) | 2002/04 - 2014 | 545 participants with ID, ≥16 years of age | 47.0-57.8%,  p<0.001 | 23.5-26.1%, p>0.05 | / | / | / | 4.6-9.4%,  p<0.001 | / | 9.9-22.0%,  p<0.001 | 1. Antipsychotics  2. Antidepressants  3. Hypnotics/ Anxiolytics | 1. Antipsychotics  2. Antidepressants  3. Hypnotics/ Anxiolytics |
| Antidepressant prescribing for adult people with an intellectual disability living in England, Branford et al. (2022) (20) | 2009/12-2021 | Psychotropic prescribing by GPs for people with IDs* | / | 17.0-17.5% |  | 9.3 (2016)-8.6% | / | / | / | 16.9-24.6% | 1. Antipsychotics  2. Antidepressants  3. Benzodiazepines | 1. Antidepressants  2. Antipsychotics  3. Benzodiazepines |

Abbreviations: ID- intellectual disability, GP- general practitioner; * The overall coverage across 5 years is between 56 and 60% of GPs.
